# Global consensus for the screening, diagnosis, staging, treatment, and surveillance of ovarian cancer in areas of significant resource limitations: report from the International Gynecological Cancer Society consensus meeting

**DOI:** 10.3389/fonc.2025.1677427

**Published:** 2026-01-12

**Authors:** Fernando Maluf, Henrique A. Helber, Francinne Tostes, Ligia A. Maluf, Juliana K. Helito, Renato Moretti-Marques, Graziela Z. Dal Molin, Bruno Roberto B. Azevedo, David I. Ortiz, David Cantu, Agnaldo L. Silva Filho, Angelica Nogueira-Rodrigues, Reitan Ribeiro, Georgia F. Cintra, Gustavo Guitmann, Andreia Cristina de Melo, Eduardo Paulino, Glauco Baiocchi, Leandro Resende, Michelle S. de Almeida, Diocesio A. de Andrade, Julio L. de la Vega, Florencia Noll, Fabio Fin, Andre Lopes, Miguel E. Correa, Joseph S. Ng, Daniel Sanabria, Gabriel Rendon, Fernando Heredia, Ana Paula E. Lopes, Myriam B. Mussi, Marcin Bobinski, Milagros P. Quintanilla, Jimena Meymar, David Atallah, Audrey Tsunoda

**Affiliations:** 1 Hospital Israelita Albert Einstein, São Paulo, Brazil; 2 Beneficencia Portuguesa de Sao Paulo, São Paulo, Brazil; 3 Oncoclinicas do Brasil, São Paulo, Brazil; 4 Sociedad Medica del Instituto Nacional de Cancerologia, Mexico City, Mexico; 5 Universidade Federal de Minas Gerais, Belo Horizonte, Brazil; 6 McGill University, Montreal, QC, Canada; 7 Instituto Brasileiro de Controle do Cancer, São Paulo, Brazil; 8 Instituto Nacional de Cancer, Rio de Janeiro, Brazil; 9 A C Camargo Cancer Center, São Paulo, Brazil; 10 Hospital do Coracao, São Paulo, Brazil; 11 Gynecare Womens Health Clinic, Guatemala City, Guatemala; 12 Sanatorio Allende Ginecologia y Obstetricia, Córdoba, Argentina; 13 Centro de Oncologia do Parana Ltda, Curitiba, Brazil; 14 Onco Prevee, Lima, Peru; 15 National University Cancer Institute, Singapore, Singapore; 16 Fundacion Universitaria de Ciencias de la Salud, Bogotá, Colombia; 17 Instituto de Cancerologia, Medellín, Colombia; 18 Instituto Oncologico Fundacion Arturo Lopez Perez, Providencia, Chile; 19 Sultan Qaboos Comprehensive Cancer Care & Research Center, Muscat, Oman; 20 Hospital Italiano de Buenos Aires, Buenos Aires, Argentina; 21 Independent Translational Medicine Laboratory, Department of Medical Genetics, Medical University in Lublin, Lublin, Poland; 22 Asociacion Medica del Centro Medico ABC, Mexico City, Mexico; 23 Kenes Israel, Lod, Israel; 24 Hôtel Dieu de France, Saint Joseph University, Beirut, Lebanon; 25 HCor, Ecomedical Center, Hospital Erasto Gaertner, PUCPR, Curitiba, Brazil

**Keywords:** ovarian cancer, resource-stratified guidelines, low- and middle-income countries, global health, consensus statement

## Abstract

**Introduction:**

The objective of this consensus report is to provide practical, resource-stratified recommendations for the management of ovarian cancer in countries with limited resources, where direct application of international guidelines is often difficult. To address these challenges, recommendations were developed from a panel of international experts.

**Methods:**

The panel met in New York in September/October 2022 during the International Gynecological Cancer Society congress and was composed of specialists from countries in Africa, Asia, Eastern Europe, Latin America, and the Middle East. The panel addressed 72 questions and provided recommendations for the diagnosis, staging, treatment, and surveillance of ovarian cancer. Consensus was defined as at least 75% of the voting members selecting a particular recommendation, whereas a majority vote was considered when one option garnered between 50% and 74.9% of votes. Resource limitation was defined as any issues limiting access to qualified surgeons, contemporary imaging or radiation-oncology techniques, antineoplastic drugs, or funding for providing modern medical care.

**Results:**

Consensus was reached for 10 out of 72 (13.9%) questions presented to the panel, whereas a majority vote was reached for 26 (36.1%) additional questions. For the remaining questions, there was considerable heterogeneity in the recommendations.

**Conclusion:**

The development of guidelines focusing on areas facing more severe resource limitations may improve medical practice and patient care.

## Introduction

Epithelial ovarian cancer is the leading cause of death among gynecologic cancers, with approximately 324.635 new cases and 207.252 deaths worldwide in 2022. It is the 8th most common cancer among women, accounting for about 3.6% of all female cancers in 2022, and studies show it remains the second most common gynecologic cancer in developing countries, after cervical cancer. The highest estimated incidence rates are still observed in Europe (1, 2).

Ovarian cancer usually has a poor prognosis, and late diagnosis is the main cause of this mortality. It lacks early detection or effective screening tests and typically presents at a late stage, when the 5-year relative survival rate is only 30% (3).

Optimal surgery and platinum-based chemotherapy remain the mainstay of treatment. Standard chemotherapy combines carboplatin and paclitaxel. Bevacizumab, an antiangiogenic therapy, is an option in front and later lines of treatment combined with chemotherapy and maintenance, especially in advanced stages or when optimal cytoreduction was not feasible due to tumor location and volume. Recent studies show that PARP (PolyADP-ribose) polymerase) inhibitors are an important maintenance strategy, mainly in homologous recombination-deficient tumors (including BRCA1/2 mutated) (4).

In countries with limited resources, cancer care quality is heterogeneous, and practitioners often face challenges beyond those in more affluent regions. Implementing international guidelines is difficult in resource-limited settings, as most originate from North America and Western Europe. One alternative for developing countries is to follow adapted or stratified guidelines from prominent organizations, such as the National Comprehensive Cancer Network (NCCN) (5). Another option is to develop local guidelines and consensus panels, as we pursued in this consensus. We leveraged a large international meeting to organize a panel that could provide consensus recommendations on topics relevant to ovarian cancer in limited-resource settings. This article is part of a series of reports from that meeting, convened under the auspices of the International Gynecological Cancer Society (6).

## Methods

### Composition, organization, and objectives of the panel

A 15-member steering committee developed the 72 questions after conducting a targeted literature review, evaluating major international guidelines (including NCCN and ESMO), and incorporating expert proposals. The preliminary question set was refined through iterative discussion within the committee before being submitted for voting. All panelists completed conflict-of-interest disclosures prior to participation. The panel was composed of invited specialists in gynecological oncology from 10 countries: Argentina, Brazil, Chile, Colombia, Guatemala, Israel, Lebanon, Mexico, Oman, and Poland. These members were opinion leaders in gynecology, oncological surgery, medical oncology, and radiation oncology in their respective countries. Using an electronic voting system, the panel answered a single structured questionnaire in polling sessions held on 30 September and 1 October 2022 during the International Gynecological Cancer Society congress in New York, USA. The questionnaire contained 72 questions, of which 23 related to diagnosis and genetic evaluation, 18 to surgical management, 16 to systemic treatment (first-line and recurrent settings), 8 to maintenance strategies, and 7 to staging and surveillance. Consensus was predefined as ≥75% agreement, consistent with thresholds commonly applied in consensus frameworks such as the RAND/UCLA Appropriateness Method and used in multiple ESMO consensus conferences. Responses between 50% and 74.9% were classified as majority vote without consensus (7).

One polling session with multiple-choice questions was scheduled for each main topic that forms the subheadings described below. When answering, panelists were instructed to assume that recommended interventions were approved, available, and had no contraindications in the described scenario. The staging classification used was the most recent from the International Federation of Obstetrics and Gynecology (7).

### Definition of resource limitation

The World Bank classifies economies into four groups based on average income: high, upper-middle, lower-middle, and low (8). Although panel members came from countries in different income groups, the socioeconomic framework used here relates to the availability of ideal resources. This is particularly relevant in countries with heterogeneous healthcare systems. Regardless of individual national situations, this work focuses on “areas” rather than “countries,” acknowledging that medical practice may be limited in certain settings despite a country’s overall resources. Resource limitation was broadly defined as restricted access to qualified surgeons, modern imaging or radiation techniques, antineoplastic drugs, or overall funding for state-of-the-art care.

### Statistical analysis

Results for each question addressed by the panel are presented descriptively and grouped by clinical setting or issue. If at least 75% of voting members selected a specific option, consensus was reached. If 50%–74.9% selected an option, it was considered a majority vote without consensus.

***In accordance with the journal’s guidelines, we will provide our data for independent analysis by a selected team by the Editorial Team for the purposes of additional data analysis or for the reproducibility of this study in other centers if such is requested.***

## Results

### Section 1: diagnosis and genetic evaluation

There was no consensus for any of the four questions related to diagnosis and genetic evaluation (**Supplementary Table S1**). Regarding the methodology of pursuing the *BRCA* test, the majority of the panel voted for only testing for germline mutations (53%), followed by only testing for somatic mutations (32%). There was no consensus or majority votes for the questions about the minimum exams to diagnose ovarian cancer.

### Section 2: surgical considerations

Eleven questions were asked about surgical treatment (**Supplementary Table S2**). Consensus was present for only one question. For patients with advanced disease (stages III or IV) and not candidates for optimal primary cytoreduction, the main treatment approach would be platinum-based chemotherapy, for 3 to 4 cycles, and if any clinical response, laparotomy for interval cytoreductive surgery, followed by more 3 to 4 cycles of platinum-based chemotherapy for 91% of the panelists.

In addition, a majority vote was present for five of 11 questions. The minimum acceptable facility infrastructures and/or health care team for surgery in patients with presumed advanced stage (III or IV), ovarian cancer was an intensive care unit and gynecologic oncology-trained surgeon (68%). The main surgical goals were diagnosis, staging and cytoreduction (67%). The main surgical procedures for surgical staging in presumed early-stage ovarian cancer when the surgeons do not have full training in oncology were total abdominal hysterectomy (TAH) and bilateral salpingoophorectomy (BSO) plus peritoneal cytology plus omentectomy plus peritoneal surface evaluation, with no lymph node assessment (64%). In the case of peritoneal disease (stage IIB or IIIB-C disease), the main surgical objective was cytoreductive surgery with no macroscopic residual disease (69%). Finally, in case of peritoneal disease (stage IIB or IIIB-C disease), the main surgical findings that limit and contraindicate the primary cytoreductive when the surgeons do not have full training in oncology was mesenteric retraction or hepatic hilus involvement or requirement of extensive bowel resection (64%).

There was generally considerable heterogeneity in the responses for the other eight additional questions.

### Section 3: first-line systemic treatment

Fourteen questions were asked about first-line systemic treatment (**Supplementary Table S3**). Consensus was achieved for only one question. The minimum acceptable number of chemotherapy cycles for adjuvant treatment in stage III or IV ovarian cancer in areas of severe resource limitations is six cycles for 82% of the panelists.

In addition, a majority vote was present for six of 14 questions. The minimum acceptable chemotherapy regimen for adjuvant treatment in stage II ovarian cancer patients with no cisplatin contra-indication in areas of severe resource limitations is carboplatin and paclitaxel every 3 weeks (56%). The minimum acceptable number of chemotherapy cycles for adjuvant treatment in stage II ovarian cancer patients in areas of severe resource limitations is six (71%). The minimum acceptable chemotherapy regimen for adjuvant treatment in stage III or IV ovarian cancer patients with no cisplatin contra-indication in areas of severe resource limitations is carboplatin and paclitaxel every 3 weeks (72%). The minimum acceptable chemotherapy regimen for adjuvant treatment in stage III or IV ovarian cancer patients with important comorbidities and/or cisplatin contra-indication in areas of severe resource limitations is carboplatin and paclitaxel every 3 weeks (56%). The minimum acceptable chemotherapy regimen for neoadjuvant treatment in stage III or IV ovarian cancer patients with no cisplatin contra-indication in areas of severe resource limitations is carboplatin and paclitaxel every 3 weeks (59%). Finally, the minimum acceptable number of chemotherapy cycles for neoadjuvant treatment in stage III or IV ovarian cancer patients prior to surgery in areas of severe resource limitations is three (70%).

For the six additional questions, there was generally considerable heterogeneity in the responses.

### Section 4: maintenance treatment

Only two questions were asked about first-line maintenance treatment (**Supplementary Table S4**) with no consensus. Regarding PARP inhibitors being prescribed in areas with limited resources after first-line chemotherapy, 48% of the panelists voted never and 26% only in BRCA mutated patients after response to platinum-based chemotherapy. While in the platinum-sensitive recurrence setting 47% of the panelists voted never and 23% only in BRCA mutated and/or HRD (Homologous Recombination Deficiency) patients after response to platinum-based chemotherapy.

### Section 5: platinum-sensitive recurrence

Seven questions were asked about platinum-sensitive recurrence setting (**Supplementary Table S5**). Consensus was present for none of those questions.

However, a majority vote was present in two questions. After the diagnosis of peritoneal recurrence in a platinum-sensitive setting, the first approach in areas of severely limited resources for patients with at least one of the following characteristics: ECOG 2 or more or severe and/or comorbidities, should be platinum-based chemotherapy for a total of 6 cycles followed by surveillance for 50% of the panelists. The minimum acceptable number of chemotherapy cycles for recurrent platinum-sensitive ovarian cancer patients in areas of severe resource limitations is six cycles for 58%.

For the five additional questions, there was generally considerable heterogeneity in the responses.

### Section 6: platinum resistant recurrence

Ten questions were asked about platinum-resistant recurrence setting (**Supplementary Table S6**). Consensus was present for none of those questions.

However, a majority vote was present for five questions. After the diagnosis of peritoneal recurrence in a platinum-resistant setting (PFS <6 months), the first approach in areas of severely limited resources (ECOG 0 or 1) should be non-platinum-based chemotherapy followed by surveillance for 58% of the panelists. In case of diagnosis of bowel obstruction due to peritoneal implants in platinum-resistant disease, and without effective response to clinical treatment, the first approach in areas of severely limited resources (ECOG 2 or more or severe and/or comorbidities) should be supportive care for 68%. The minimum acceptable salvage chemotherapy regimen for platinum-resistant/refractory ovarian cancer patients with severe comorbidities not exposed to taxanes in areas of severe resource limitations should be weekly paclitaxel for 60%. The minimum acceptable number of chemotherapy cycles for recurrent platinum-resistant/refractory ovarian cancer patients in areas of severe resource limitation should be 6 cycles for 66% and for women with advanced, recurrent, and platinum-resistant/refractory ovarian cancer with no clinical trial available, the recommendation of best supportive care in an area with limited resources should be based on performance status > 2, unrelated to the line of treatment for 55%.

For the five additional questions, there was generally considerable heterogeneity in the responses.

### Section 7: WHO essential medicines list

One question was asked about eight WHO essential 8 medicines (**Supplementary Table S7**).

The question was “Which would you consider as appropriate treatment options for women with metastatic ovarian cancer in the setting of limited healthcare resources since you can purchase them at an affordable price from the generic manufacturer? ‘‘

Consensus “yes’’ was present for 2 medicines: doxorubicin (90%) and gemcitabine (89%).

Majority “yes’’ was present for 2 medicines: topotecan (69%) and cyclophosphamide (64%).

Majority “no’’ was present for 3 medicines: ifosfamide (65%), 5FU (73%) and vinorelbine (50%).

### Section 8: symptoms management

Five questions were asked about symptom management (**Supplementary Table S8**). Consensus was present for only one question. The most adequate opioid agent to manage moderate to severe cancer pain in patients with platinum-refractory advanced ovarian cancer after multiple lines of systemic therapy in areas of severe resource limitations is morphine (81%). A majority vote was present for one question. The most adequate initial treatment strategy to manage malignant bowel obstruction secondary to peritoneal carcinomatosis in patients with platinum-refractory advanced ovarian cancer after multiple lines of systemic therapy in areas of severe resource limitations is nasogastric tube (52%).

### Section 9: staging and follow-up

Eleven questions were asked about staging and follow-up (**Supplementary Table S9**). Consensus was present for three questions. The minimum acceptable staging tools in patients with clinical stage III or IV disease in an area with severely limited resources are operative staging, CA125, pelvic and abdominal ultrasound, and chest X-ray (87%). The minimum acceptable frequency of follow-up for stage III or IV ovarian cancer patients after curative treatment in an area with severe resource limitations is every 3 months in the first 2 years, after that, every six months until 5 years from the end of the treatment (85%), and the minimum acceptable frequency of follow-up for stage III or IV recurrent ovarian cancer patients after response to therapy in an area with severe resources limitations is every 3 months in the first 2 years, after that, every six months until 5 years from the treatment (77%).

A majority vote was present for three questions. The minimum acceptable staging tools in patients with clinical stage I or II disease in an area with severely limited resources are operative staging, CA125, pelvic and abdominal ultrasound, and chest X-ray (71%). The minimum acceptable frequency of follow-up for stage I or II ovarian cancer patients after curative treatment in an area with severe resource limitations is every 3 months in the first 2 years, after that, every six months until 5 years from the treatment (61%), and the minimum acceptable tools for follow-up in patients with recurrent ovarian cancer patients after response to therapy in an area with severely limited resources are clinical examination and Ca125 (56%).

## Discussion

Ovarian cancer remains difficult to treat, and management continues to evolve quickly in high-resource settings. In low- and middle-income countries, however, limited access to specialists, imaging, systemic therapy, and genetic testing creates major challenges. In these regions, practical and realistic guidelines are essential to improve outcomes. This consensus represents, to our knowledge, the first international effort to develop recommendations specifically for ovarian cancer care in settings with severe resource limitations.

Although consensus was reached for only 10 of the 72 questions (13.9%), and a majority vote for another 26 (36.1%), this variability reflects real differences in healthcare systems, access to treatment, and clinical practice across participating countries. It also likely reflects differences in the professional backgrounds of panelists, which included surgical oncologists, medical oncologists, radiation oncologists and gynecologic oncologists. These factors naturally lead to different perspectives on diagnosis and treatment. In addition, unequal geographic representation may have influenced the results, as some regions with greater limitations or different clinical practices were more represented than others. This imbalance, combined with the varied subspecialty backgrounds of participants, may help explain why consensus was not achieved for several questions (9).

Despite these differences, several areas of agreement emerged. The panel’s recommendations align with principles found in resource-stratified frameworks such as those from NCCN, including prioritizing platinum-based chemotherapy (10, 11), using cytoreductive surgery when feasible, and relying on basic imaging tools when advanced modalities are not available (12–14). These points of overlap suggest that some treatment standards can be maintained even in limited-resource settings.

Important differences were also observed. Unlike the NCCN recommendations, routine BRCA testing and PARP inhibitor maintenance were not broadly endorsed by the panel due to limited availability and high cost in many participating regions. As a result, the guidance presented here is grounded in feasibility and real-world access rather than ideal recommendations designed for high-income countries (15–17).

Regional studies support these findings, showing wide variation in access to essential oncology services and treatment options in low- and middle-income settings (9, 18). Even with this variation, developing adapted guidelines may help reduce practice gaps and support more consistent care. Future updates will be needed as international societies revise their general recommendations and as access to diagnostics and treatments improves over time.

## Data Availability

The original contributions presented in the study are included in the article/**Supplementary Material**. Further inquiries can be directed to the corresponding author.

## References

[1] Ferlay J Colombet M Soerjomataram I Parkin DM Piñeros M Znaor A . Cancer statistics for the year 2020: An overview. Int J Cancer. (2021) 149:778. doi: 10.1002/ijc.33588, PMID: 33818764

[2] Bray F Ferlay J Siegel RL Laversanne M Soerjomataram I Jemal A . Global cancer statistics 2022: GLOBOCAN estimates of incidence and mortality worldwide for 36 cancers in 185 countries. CA: A Cancer J Clin. (2024), 74. doi: 10.3322/caac.21834, PMID: 38572751

[3] Santos M de O Lima FCdSd Martins LFL Oliveira JFP Almeida LMd Cancela M de C . Estimativa de Incidência de Câncer no Brasil, 2023-2025. Rev Bras Cancerol. (2023) 69:e–213700.

[4] Sambasivan S . Epithelial ovarian cancer: Review article. Cancer Treat Res Commun. (2022) 33. doi: 10.1016/j.ctarc.2022.100629, PMID: 36127285

[5] NCCN . Clinical Practice Guidelines in Oncology (NCCN Guidelines^®^) for Guideline Ovarian Cancer/Fallopian Tube Cancer/Primary Peritoneal Cancer V.3.2023. ^©^ . National Comprehensive Cancer Network, Inc.

[6] Maluf FC Zibetti GDM Paulino E de Melo AC Racy D Ferrigno R . Recommendations for the treatment of vulvar cancer in settings with limited resources: Report from the International Gynecological Cancer Society consensus meeting. Front Oncol. (2022) 12:928568. doi: 10.3389/fonc.2022.928568, PMID: 36203438 PMC9530794

[7] Berek JS Renz M Kehoe S Kumar L Friedlander M . Cancer of the ovary, fallopian tube, and peritoneum: 2021 update. Int J Gynecol Obstet. (2021) 155:61–85. doi: 10.1002/ijgo.13878, PMID: 34669199 PMC9298325

[8] The World Bank . Classifying countries by income . Available online at: https://datatopics.worldbank.org/world-development-indicators/stories/the-classification-of-countries-by-income.html (Accessed January 17, 2024).

[9] Fundytus A Sengar M Lombe D Hopman W Jalink M Gyawali B . Access to cancer medicines deemed essential by oncologists in 82 countries: an international, cross-sectional survey. Lancet Oncol. (2021) 22:1367–77. doi: 10.1016/S1470-2045(21)00463-0, PMID: 34560006 PMC8476341

[10] Bertelsen K Grenman S Rustin GJ . How long should first-line chemotherapy continue? Ann Oncol. (1999) 10 Suppl 1:17–20. doi: 10.1023/a:1008399132535, PMID: 10219448

[11] Monk BJ Randall LM Grisham RN . The evolving landscape of chemotherapy in newly diagnosed advanced epithelial ovarian cancer. Am Soc Clin Oncol Educ Book. (2019) 39):e141–51. doi: 10.1200/edbk_239007, PMID: 31099631

[12] Neff RT Senter L Salani R . BRCA mutation in ovarian cancer: testing, implications and treatment considerations. Ther Adv Med Oncol. (2017) 9:519–31. doi: 10.1177/1758834017714993, PMID: 28794804 PMC5524247

[13] Dusic EJ Theoryn T Wang C Swisher EM Bowen DJ EDGE Study Team . Barriers, interventions, and recommendations: Improving the genetic testing landscape. Front Digit Health. (2022) 4:961128. doi: 10.3389/fdgth.2022.961128, PMID: 36386046 PMC9665160

[14] Calabrich AF Assad DX Dal-Molin G Melo AC Nogueira-Rodrigues A Kalil K . PARP inhibitors as first-line maintenance therapy in ovarian cancer: recommendations from an expert panel from Brazil. Braz J Oncol. (2023) 19:e–20230400. doi: 10.1055/s-00059887

[15] Fagotti A Ferrandina MG Vizzielli G Pasciuto T Fanfani F Gallotta V . Randomized trial of primary debulking surgery versus neoadjuvant chemotherapy for advanced epithelial ovarian cancer (SCORPION-NCT01461850). Int J Gynecol Cancer. (2020) 30:1657–64. doi: 10.1136/ijgc-2020-001640, PMID: 33028623

[16] Coleridge SL Bryant A Kehoe S Morrison J . Neoadjuvant chemotherapy before surgery versus surgery followed by chemotherapy for initial treatment in advanced ovarian epithelial cancer. Cochrane Database Syst Rev. (2021):CD005343. doi: 10.1002/14651858.CD005343.pub6, PMID: 34328210 PMC8406953

[17] Fader AN Rose PG . Role of surgery in ovarian carcinoma. J Clin Oncol. (2007) 25:2873–83. doi: 10.1200/JCO.2007.11.0932, PMID: 17617518

[18] Murphy PB Bechmann S Barrett MJ . Morphine. In: StatPearls. StatPearls Publishing, Treasure Island (FL (2024).

