# Supplementary material for: Global consensus for the screening, diagnosis, staging, treatment, and surveillance of ovarian cancer in areas of significant resource limitations: report from the International Gynecological Cancer Society consensus meeting
Source: Front Oncol. 2026 Jan 12;15:1677427. doi: 10.3389/fonc.2025.1677427 (PMC12833755; doi:10.3389/fonc.2025.1677427)
Supplement: Supplementary file 1 [file DataSheet1.pdf]

Supplementary Materials for "GLOBAL CONSENSUS FOR THE SCREENING, DIAGNOSIS, STAGING, TREATMENT, AND SURVEILLANCE OF OVARIAN CANCER IN AREAS OF SIGNIFICANT RESOURCE LIMITATIONS: REPORT FROM THE INTERNATIONAL GYNECOLOGICAL CANCER SOCIETY CONSENSUS MEETING ''

Supplementary Table 1 . Diagnosis and genetic evaluation of ovarian cancer

.

Questions	**Responses** (%)							
When should we test BRCA mutations in ovarian cancer in an area with severe limited resources?	Always	Never	Only if Family history for breast or ovarian cancer	Abstain	Unqualified to answer	-	-	-
	36%	18%	43%	0%	3%	-	-	-
How should we test BRCA mutations in ovarian cancer in an area with severe limited resources?	Only by germinative mutations	Only by somatic mutations	Abstain	Unqualified to answer	-	-	-	-
	53%	32%	9%	6%	-	-	-	-
What are the minimum exam results to diagnose ovarian cancer in an area of	Surgical laparotomy and biopsy	Ultrasound guided biopsy	Ultrasound + Ca125 findings	Computed tomography (if available)	1+3	1+4	Abstain	Unqualified to answer

limited resources?patients with suspected metastatic vulvar cancer?				+ Ca125 findings				
	20%	3%	17%	17%	13%	30%	0%	0%
What are the minimum exam results to diagnose ovarian cancer in an area of limited resources if computed tomography is not available?	Surgical laparotomy and biopsy	Ultrasound guided biopsy	Ultrasound + Ca125 findings	1 + 3	Abstain	Unqualified to answer	-	-
	14%	20%	17%	49%	0%	0%	-	-

Supplementary Table 2: Surgical considerations

Question	Responses (%)									
What is the minimum acceptable facility infrastructures and/or health care team for surgery in patients with presumed early stage (I-II), ovarian cancer in an area with severe limited resources?	Intensive Care Unit	Gynecologic oncology trained surgeon	General gynecologist or general surgeon	1 + 2	1 + 3	Abstain	Unqualified to answer	-	-	-
	3%	24%	15%	26%	32%	0%	0%	-	-	-
What is the minimum acceptable facility infrastructures and/or health care team for surgery in patients with presumed early stage (III-IV), ovarian cancer in an	Intensive Care Unit	Gynecologic oncology trained surgeon	General gynecologist or general surgeon	1 + 2	1 + 3	Abstain	Unqualified to answer	-	-	-
	3%	6%	0%	68%	23%	0%	0%	-	-	-

area with severe limited resources?										
What are the main surgical goals in a severe limited resource setting?	Diagnosis	Staging	Cytoreduction	1 + 2	1 + 2 + 3	Abstain	Unqualified to answer	-	-	-
	12%	0%	15%	6%	67%	0%	0%	-	-	-
What are the main surgical procedures for surgical staging in presumed early stage ovarian cancer in a severe limited resource setting?	Total Abdominal Hysterectomy (TAH) and Bilateral Salpingoophorectomy (BSO)	TAH + BSO + Peritoneal Cytology + Omentectomy + Peritoneal surface evaluation	2 + Pelvic lymph node sampling	2 + Pelvic and para-aortic lymph node sampling	2 + Systematic pelvic and paraaortic lymph node dissection up to the renal vessels	Abstain	-	-	-	-
	3%	43%	3%	28%	20%	0	-	-	-	-

What are the main surgical procedures for surgical staging in presumed early stage ovarian cancer in a severe limited resource setting when the surgeons do not have a full training in oncology?	Total Abdominal Hysterectomy (TAH) and Bilateral Salpingoophorectomy (BSO)	TAH + BSO + Peritoneal Cytology + Omentectomy + Peritoneal surface evaluation	2 + Pelvic lymph node sampling	2 + Pelvic and para-aortic lymph node sampling	2 + Systematic pelvic and paraaortic lymph node dissection up to the renal vessels	Abstain	-	-	-	-
	4%	64%	12%	4%	12%	4%	-	-	-	-
In case of peritoneal disease (stage IIB or IIIB-C disease), what should be the main surgical objective in severe limited resource setting?	Total Abdominal Hysterectomy (TAH) and Bilateral Salpingoophorectomy (BSO) and	Cytoreductive surgery with the objective of residual disease of ≤2cm in the greatest dimension	Cytoreductive surgery with the objective of residual disease of ≤1cm	Cytoreductive surgery with the objective of no macroscopic residual disease	Abstain	Unqualified to answer	-	-	-	-

	biopsy of the suspicious implant. Residual disease of >2cm left										
	7%	7%	17%	69%	0%	0%	-	-	-	-	
In case of peritoneal disease (stage IIB or IIIB-C disease), what should be the main surgical objective in severe limited resource setting when the surgeons do not have a full training in oncology?	Total Abdominal Hysterectomy (TAH) and Bilateral Salpingoophorectomy (BSO) and biopsy of the	Cytoreductive surgery with the objective of residual disease of ≤2cm in the greatest dimension	Cytoreductive surgery with the objective of residual disease of ≤1cm	Cytoreductive surgery with the objective of no macroscopic residual disease	Do not procedure surgery if not able to achieve complete cytoreduction	Abstain	Unqualified to answer	-	-	-	

	suspicious implant. Residual disease of >2cm left									
	13%	10%	17%	10%	47%	0%	3%	-	-	-
In case of peritoneal disease (stage IIB or IIIB-C disease), what are the main surgical findings that limit and contraindicate the primary cytoreductive surgery in areas of severe limited resources (ECOG 0-1)?	Presence of any peritoneal disease	Presence of any bowel implants	Presence of diaphragmatic implants	Presence of implants that require any type of bowel resection	Presence of any upper abdomen disease (excluding the greater	Presence of implants regardless of size in all abdominal quadrants	Presence of high disease volume implicating in high complexity	Mesenteric retraction or hepatic hilus involvement or requirement of extensive bowel	Abstain	Unqualified to answer

								resection		
	4%	0%	0%	7%	4%	0%	44%	30%	4%	7%
In case of peritoneal disease (stage IIB or IIIB-C disease), what are the main surgical findings that limit and contraindicate the primary cytoreductive surgery in areas of severe limited resources (ECOG 2 or more and/or severe comorbidities)?	Presence of any peritoneal disease	Presence of any bowel implants	Presence of diaphragmatic implants	Presence of implants that require any type of bowel resection	Presence of any upper abdomen disease (excluding the greater	Presence of implants regardless of size in all abdominal quadrants	Presence of high disease volume implicating in high complexity	Mesenteric retraction or hepatic hilus involvement or requirement of extensive bowel resection	Abstain	Unqualified to answer

	16%	0%	0%	3%	7%	3%	42%	10%	13%	6%
In case of peritoneal disease (stage IIB or IIIB-C disease), what are the main surgical findings that limit and contraindicate the primary cytoreductive surgery in areas of severe limited resources (ECOG 0-1) when the surgeons do not have a full training in oncology?	Presence of any peritoneal disease	Presence of any bowel implants	Presence of diaphragmatic implants	Presence of implants that require any type of bowel resection	Presence of any upper abdomen disease (excluding the greater	Presence of implants regardless of size in all abdominal quadrants	Presence of high disease volume implicating in high complexity	Mesenteric retraction or hepatic hilus involvement or requirement of extensive bowel resection	Abstain	Unqualified to answer
	3%	0%	3%	9%	9%	6%	12%	49%	3%	6%

In case of peritoneal disease (stage IIB or IIIB-C disease), what are the main surgical findings that limit and contraindicate the primary cytoreductive surgery in areas of severe limited resources (ECOG 2 or more and/or severe comorbidities) when the surgeons do not have a full training in oncology?	Presence of any peritoneal disease	Presence of any bowel implants	Presence of diaphragmatic implants	Presence of implants that require any type of bowel resection	Presence of any upper abdomen disease (excluding the greater	Presence of implants regardless of size in all abdominal quadrants	Presence of high disease volume implicating in high complexity	Mesenteric retraction or hepatic hilus involvement or requirement of extensive bowel resection	Abstain	Unqualified to answer
	17%	0%	5%	0%	3%	0%	3%	64%	5%	3%

Supplementary Table 3. Questions related to the first line syatemic treatment.

Question	Responses (%)								
When primary cytoreduction is not feasible for advanced stages (stages III or IV disease), what should be the treatment approach in areas of severe limited resources (ECOG 0- 1)?	Platinum based chemotherapy, 6 cycles, and surveillance after clinical response	Platinum based chemotherapy, 6 cycles. If complete clinical response is not achieved, send to palliative care	Platinum based chemotherapy, 6 cycles. If complete clinical response is not acchieved, change to nonplatinum chemotherapy	Platinum based chemotherapy, 6 cycles. If complete clinical response is not achieved, deliver 3 more cycles	Platinum based chemotherapy, 3-4 cycles. If any clinical response, laparotomy for interval cytoreductive surgery, followed by more 3-4 cycles of platinum based chemotherapy	Platinu m based chemoth erapy, 6 cycles. If any clinical respons e, laparoto my for cytoredu ctive surgery.	Abstain	Unqualif ied to answer	-
	0%	0%	0%	0%	91%	3%	3%	3%	-

When primary cytoreduction is not feasible for advanced stages (stages III or IV disease), what should be the treatment approach in areas of severe limited resources (ECOG 2 or more or Age >75y or clinically fragile patient and/or severe comorbidities)?	Platinum based chemotherapy, 6 cycles, and surveillance after clinical response	Platinum based chemotherapy, 6 cycles. If complete clinical response is not achieved, send to palliative care	Platinum based chemotherapy, 6 cycles. If complete clinical response is not acchieved, change to nonplatinum chemotherapy	Platinum based chemotherapy, 6 cycles. If complete clinical response is not achieved, deliver 3 more cycles	Platinum based chemotherapy, 3-4 cycles. If any clinical response, laparotomy for interval cytoreductive surgery, followed by more 3-4 cycles of platinum based chemotherapy	Platinum based chemotherapy, 6 cycles. If any clinical response, laparotomy for cytoreductive surgery.	Abstain	Unqualified to answer	-
	17%	12%	0%	0%	42%	21%	8%	0%	-
What is the minimum acceptable chemotherapy regimen for adjuvant treatment in stage I high-risk ovarian cancer patients with no	Cisplatin alone	Carboplatin alone	Cisplatin and paclitaxel every 3 weeks	Carboplatin and paclitaxel every 3 weeks	Carboplatin weekly and paclitaxel weekly	Abstain	Unqualified to answer	-	-

cisplatin contra-indication in areas of severe resources limitations?	3%	14 %	28%	47%	5%	0%	3%	-	-
What is the minimum acceptable chemotherapy regimen for adjuvant treatment in stage I high-risk ovarian cancer patients with important comobordities and/or cisplatin contra-indication in areas of severe resources limitations?	Carboplatin alone	Carboplatin and paclitaxel every 3 weeks	Carboplatin weekly and paclitaxel weekly	No platinum regimen	Abstain	Unqualified to answer-	-	-	-
	29%	40%	11%	11%	3%	6%	-	-	-
. What is the minimum acceptable number of chemotherapy cycles for adjuvant treatment in stage I high-risk ovarian cancer patients in areas of severe resources limitations?	One	Two	Three	Four	Five	Six	Abstain	Unqualified to answer	-
	0%	0%	40%	15%	0%	36%	0%	9%	-
What is the minimum acceptable chemotherapy regimen for adjuvant treatment in stage II ovarian cancer	Cisplatin alone	Carboplatin alone	Cisplatin and paclitaxel every 3 weeks	Carboplatin and paclitaxel every 3 weeks	Carboplatin weekly and	Abstain	Unqualified to answer	-	-

patients with no cisplatin contra-indication in areas of severe resources limitations?					paclitaxel weekly				
	0%	6%	23%	56%	6%	0%	9%		-
What is the minimum acceptable chemotherapy regimen for adjuvant treatment in stage II ovarian cancer patients with important comorbidities and/or cisplatin contraindication in areas of severe resources limitations?	Carboplatin alone	Carboplatin and paclitaxel every 3 weeks	Carboplatin weekly and paclitaxel weekly	No platinum regimen	Abstain	Unqualified to answer-	-	-	-
	25%	44%	9%	3%	0%	19%	-	-	-
What is the minimum acceptable number of chemotherapy cycles for adjuvant treatment in stage II ovarian cancer patients in areas of severe resources limitations?	One	Two	Three	Four	Five	Six	Abstain	Unqualified to answer	-
	0%	0%	6%	14%	0%	71%	0%	9%	-
What is the minimum acceptable chemotherapy regimen for adjuvant treatment in stage III-IV ovarian	Cisplatin alone	Carboplatin alone	Cisplatin and paclitaxel every 3 weeks	Carboplatin and paclitaxel every 3 weeks	Carboplatin weekly and	Abstain	Unqualified to answer	-	-

cancer patients with no cisplatin contra-indication in areas of severe resources limitations?					paclitaxel weekly				
	0%	0%	14%	72%	0%	0%	14%	-	--
What is the minimum acceptable chemotherapy regimen for adjuvant treatment in stage III-IV ovarian cancer patients with important comorbidities and/or cisplatin contra-indication in areas of severe resources limitations?	Carboplatin alone	Carboplatin and paclitaxel every 3 weeks	Carboplatin weekly and paclitaxel weekly	No platinum regimen	Abstain	Unqualified to answer-	-	-	-
	16%	56%	9%	0%	0%	19%	-	-	-
What is the minimum acceptable number of chemotherapy cycles for adjuvant treatment in stage III-IV ovarian cancer patients in areas of severe resources limitations?	One	Two	Three	Four	Five	Six	Abstain	Unqualified to answer	-
	0%	0%	6%	9%	0%-	82%	0%	9%	-
What is the minimum acceptable chemotherapy regimen for neoadjuvant treatment in stage III-	Cisplatin alone	Carboplatin alone	Cisplatin and paclitaxel every 3 weeks	Carboplatin and paclitaxel every 3 weeks	Carboplatin weekly and	Abstain	Unqualified to answer	-	-

IV ovarian cancer patients with no cisplatin contra-indication in areas of severe resources limitations?					paclitaxel weekly				
	3%	3%	23%	59%	0%	0%-	12%	-	-
What is the minimum acceptable number of chemotherapy cycles for neoadjuvant treatment in stage III-IV ovarian cancer patients prior to surgery in areas of severe resources limitations?	One	Two	Three	Four	Five	Six	Abstain	Unqualified to answer	-
	0%	3%	70%	9%	3%	6%	0%	9%	-
What is your first-line treatment for patients with advanced ovarian cancer without access to taxanes or where taxane-related costs are prohibitive?	Platinum and 5-fluorouracil	Platinum and cyclophosphamide	Platinum and doxorubicin (not liposomal)	Platinum and gemcitabine	Platinum and topotecan-	Cisplatin	Carboplatin	Abstain	Unqualified to answer
	0%	20%	3%	32%	0%	3%	16%	0%	26%
	13.5%	37.1%	46.1%	3.4%	-	-	-	-	-

Supplementary Table 4. Questions related to the first line maintenance treatment.

Question	Responses (%)							
When should PARP inhibitors be prescribed in areas with limited resources after firstline chemotherapy?	In maintenance only in BRCA mutated patients after response to platinum based chemotherapy	In maintenance only in BRCA mutated and/or HRD patients after response to platinum based chemotherapy	In maintenance in all patients after response to platinum based chemotherapy	Never	Abstain	Unqualified to answer	-	-
	26%	13%	3%	48%	0%	10%	-	-
When should PARP inhibitors be prescribed in areas with limited resources	In maintenance only in	In maintenance only in BRCA mutated and/or	In maintenance in all patients	Never	Abstain	Unqualified to answer	-	-

after salvage chemotherapy for platinum sensitive disease?	BRCA mutated patients after response to platinum based chemotherapy	HRD patients after response to platinum based chemotherapy	after response to platinum based chemotherapy					
	14%	23%	13%	47%	0%	3%	-	-

Supplementary Table 5. Questions related to platinum-sensitive recurrence setting.

Question	Answers and frequency of responses							
After the diagnosis of peritoneal recurrence in a platinum sensitive setting (PFS >6 months), what should be the first approach in areas of severe limited resources (ECOG 0-1)?	Platinum based chemotherapy, 6 cycles, followed by surveillance	Platinum based chemotherapy, 6 cycles. If complete clinical response is not achieved, deliver 3 more cycles	Secondary cytoreduction attempt followed by platinum based chemotherapy, 6 cycles	Platinum based chemotherapy, 3-4 cycles. If any clinical response, laparotomy for secondary cytoreductive surgery, followed by more 3-4 cycles of platinum based chemotherapy	Platinum based chemotherapy, 6 cycles. If any clinical response, laparotomy for cytoreductive surgery	Supportive care	Abstain	Unqualified to answer

	23%	8%	35%	19%	15%	0%	0%	0%
After the diagnosis of peritoneal recurrence in a platinum sensitive setting (PFS >6 months), what should be the first approach in areas of severe limited resources (ECOG 2 or more or severe and/or comorbidities)?	Platinum based chemotherapy, 6 cycles, followed by surveillance	Platinum based chemotherapy, 6 cycles. If complete clinical response is not achieved, deliver 3 more cycles	Secondary cytoreduction attempt followed by platinum based chemotherapy, 6 cycles	Platinum based chemotherapy, 3-4 cycles. If any clinical response, laparotomy for secondary cytoreductive surgery, followed by more 3-4 cycles of platinum based chemotherapy	Platinum based chemotherapy, 6 cycles. If any clinical response, laparotomy for cytoreductive surgery	Supportive care	Abstain	Unqualified to answer
	50%	12%	4%	15%	4%	15%	0%	0%

In case of diagnosis of bowel obstruction due to peritoneal implants in platinum sensitive disease, and without effective response to clinical treatment, what should be the first approach in areas of severe limited resources (ECOG 0-1)?	Platinum based chemotherapy and supportive care	Platinum based chemotherapy and surgery if no resolution of bowel obstruction	Palliative surgical approach followed by platinum based chemotherapy	Gastrostomy	Supportive care	Abstain	Unqualified to answer	-
	6%	25%	47%	3%	19%	0%	0%	-
In case of diagnosis of bowel obstruction due to peritoneal implants in platinum sensitive disease, and without effective response to clinical treatment, what should be the first approach in areas of	Platinum based chemotherapy and supportive care	Platinum based chemotherapy and surgery if no resolution of bowel obstruction	Palliative surgical approach followed by platinum based chemotherapy	Gastrostomy	Supportive care	Abstain	Unqualified to answer	-
	7%	7%	32%	4%	46%	4%	0%	-

severe limited resources (ECOG 2 or more or severe and/or comorbidities)?								
What is the minimum acceptable salvage chemotherapy regimen for recurrent platinum sensitive ovarian cancer patients in areas of severe resources limitations?	Cisplatin alone	Carboplatin alone	Cisplatin and paclitaxel every 3 weeks	Carboplatin and paclitaxel every 3 weeks	Carboplatin weekly and paclitaxel weekly	Platinum and gencitabin e	Carboplat in and doxorubic in (not liposomal)	Abstai n
	3%	15%	12%	35%	3%	15%	3%	0%
What is the minimum acceptable salvage chemotherapy regimen for recurrent platinum sensitive ovarian cancer patients with severe	Cisplatin alone	Carboplatin alone	Cisplatin and paclitaxel every 3 weeks	Carboplatin and paclitaxel every 3 weeks	Carboplatin weekly and paclitaxel weekly	Platinum and gencitabin e	Carboplat in and doxorubic in (not liposomal)	Abstai n

comorbidities in areas of severe resources limitations?	0%	29%	8%	25%	4%	17%	0%	0%
What is the minimum acceptable number of chemotherapy cycles for recurrent platinum sensitive ovarian cancer patients in areas of severe resources limitations?	One	Two	Three	Four	Five	Six	Abstain	Unqualified to answer
	0%	0%	9%	24%	0%	58%	0%	9%%

Supplementary Table 6. Questions related to platinum-resistant recurrence setting..

Question	Answers and frequency of responses							
After the diagnosis of peritoneal recurrence in a platinum resistant setting (PFS<6 months), what sould be the first approach in areas of severe limited resources (ECOG 0-1)?	Platinum based chemotherapy, followed by surveillance	Non-platinum based chemotherapy, followed by surveillance	Secondary cytoreduction attempt followed by chemotherapy, 6 cycles	Chemotherapy. If any clinical response, laparotomy for secondary cytoreductive surgery	Supportive care	Abstain	Unqualified to answe	-
	6%	58%	6%	15%	12%	0%	3%	-
After the diagnosis of peritoneal recurrence in a platinum resistant setting (PFS<6 months), what sould be the first approach in areas of severe limited resources	Platinum based chemotherapy, followed by surveillance	Non-platinum based chemotherapy, followed by surveillance	Secondary cytoreduction attempt followed by chemotherapy, 6 cycles	Chemotherapy. If any clinical response, laparotomy for secondary	Supportive care	Abstain	Unqualified to answe	-

(ECOG 2 or more or severe and/or comorbidties)?				cytoreductive surgery				
	3%	39%	0%	3%	48%	0%	7%	-
In case of diagnosis of bowel obstruction due to peritoneal implants in platinum resistant disease, and without effective response to clinical treatment, what should be the first approach in areas of severe limited resources (ECOG 0-1)?	Chemotherapy and supportive care	Chemotherapy and palliative surgery attempt if no resolution of bowel obstruction	Palliative surgical attempt followed by chemotherapy	Gastrostomy	Supportive care	Abstain	Unqualified to answer	-
	3%	9%	44%	6%	35%	3%	0%	-
In case of diagnosis of bowel obstruction due to peritoneal implants in platinum resistant disease, and without	Chemotherapy and supportive care	Chemotherapy and palliative surgery attempt if no	Palliative surgical attempt followed by	Gastrostomy	Supportive care	Abstain	Unqualified to answer	-

effective response to clinical treatment, what should be the first approach in areas of severe limited resources (ECOG 2 or more or severe and/or comorbidities)?		resolution of bowel obstruction	chemotherapy					
	10%	10%	9%	0%	68%	3%	0%	-
What is the minimum acceptable salvage chemotherapy regimen for recurrent platinum resistant/refractory ovarian cancer patients exposed to taxanes in areas of severe resources limitations?	Gemcitabine	Topotecan	Doxorrubicin	Cyclophosphamide	Paclitaxel weekly	Docetaxel	Abstain	Unqualified to answer
	47%	0%	28%	3%	3%	0%	3%	16%
What is the minimum acceptable salvage	Gemcitabine	Topotecan	Doxorrubicin	Cyclophosphamide	Paclitaxel weekly	Docetaxel	Abstain	Unqualified

chemotherapy regimen for recurrent platinum resistant/refractory ovarian cancer patients with severe comorbidities exposed to taxanes in areas of severe resources limitations?								to answer
	41%	9%	16%	0%	15%	0%	0%	19%
What is the minimum acceptable salvage chemotherapy regimen for platinum resistant/refractory ovarian cancer patients NOT exposed to taxanes in areas of severe resources limitations?	Gemcitabine	Topotecan	Doxorrubicin	Cyclophosphamide	Paclitaxel weekly	Docetaxel	Abstain	Unqualified to answer
	16%	0%	9%	0%	47%	3%	3%	22%
What is the minimum acceptable salvage	Gemcitabine	Topotecan	Doxorrubicin	Cyclophosphamide	Paclitaxel weekly	Docetaxel	Abstain	Unqualified

chemotherapy regimen for platinum resistant/refractory ovarian cancer patients with severe comorbidities NOT exposed to taxanes in areas of severe resources limitations?								to answer
	17%	0%	0%	0%	60%	0%	3%	20%
What is the minimum acceptable number of chemotherapy cycles for recurrent platinum resistant/refractory ovarian cancer patients in areas of severe resources limitation?	One	Two	Three	Four	Five	Six	Abstain	Unqualified to answer
	0%	0%	7%	17$	3%	66%	0%	7%
For women with advanced, recurrent, and platinum	After first-line treatment	After second-line treatment	After third-line treatment or more	Performance status > 2, unrelated to	Abstain	Unqualified to answer	-	-

resistant/refractory ovarian cancer with no clinical trial available, when do you recommend best supportive care in an area with limited resources?le?				line of treatment				
	0%	29%	10%	55%	0%	6%	-	-

.

Supplementary Table 7. WHO essential medicines list.

Question	Answers and frequency of responses							
Each of the following drugs is on the WHO essential medicines list. You are able to purchase them at an affordable price from generic manufacturer. Which would you consider as appropriate treatment options for women with metastatic ovarian cancer in the setting of limited healthcare resources?	Ifosfamide	Topotecan	Doxorubicin	Gemcitabine	Cyclophosphamide	5-FU	Vinorelbine	Etoposide
	Yes (14%)<br>No (65%)<br>Abstain (7%)<br>Unqualified to answer (14%)	Yes (69%)<br>No (21%)<br>Abstain (0%)<br>Unqualified to answer (10%)	Yes (90%)<br>No (5%)<br>Abstain (0%)<br>Unqualified to answer (5%)	Yes (89%)<br>No (0%)<br>Abstain(0%)<br>Unqualified to answer (11%)	Yes (64%)<br>No (22%)<br>Abstain (0%)<br>Unqualified to answer (14%)	Yes (14%)<br>No (73%)<br>Abstain (0%)<br>Unqualified to answer (13%)	Yes (23%)<br>No (50%)<br>Abstain (0%)<br>Unqualified to answer (27%)	Yes (48%)<br>No (36%)<br>Abstain (0%)<br>Unqualified to answer (16%)

Supplementary Table 8. Questions related to drugs used in ovarian cancer included in the World Health Organization (WHO) essential medicines list that can be purchased at an affordable price from generic manufacturers.

Supplementary Table 8. Symptoms management

Question	Answers and frequency of responses								
What is the most adequate initial treatment strategy to manage malignant bowel obstruction secondary to peritoneal carcinomatosis in patients with platinum refractory advanced ovarian cancer after multiple lines of systemic therapy in areas of severe resources limitations?	Surgery	Self-expanding stent placement	Pharmacologic management	Nasogastric tube	Endoscopic gastrostomy tube	Abstain	Unqualified to answer	-	-
	27%	3%	12%	52%	6%	0%	0%	-	-
What is the most adequate pharmacologic treatment to manage nausea and vomiting in patients with malignant bowel	A glucocorticoid	An anticolinergic agent (e.g. scopalamine) and an	A somatostatin analog and a antiemetic	An anticolinergic agent (e.g. scopalamine), an antiemetic	A somatostatin analog, an antiemetic (e.g.haloperid	Abstain	Unqualified to answer	-	-

obstruction secondary to platinum refractory advanced ovarian cancer after multiple lines of systemic therapy in areas of severe resources limitations?-		antiemetic (e.g.haloperidol)	(e.g.haloperidol)	(e.g.haloperidol) and a glucocorticoid	ol), and a glucocorticoid				
	3%	17%	10%	40%	27%	3%	0%	-	-
What is the most adequate opioid agent to manage moderate to severe cancer pain in patients with platinum refractory advanced ovarian cancer after multiple lines of systemic therapy in areas of severe resources limitations?	Fentanyl	Hydromorphone	Morphine	Buprenorphine	Meperidine	Metthadone	Abstain	Unqualified to answer	-
	10%	3%	81%	6%	0%	0%	0%	0%	-

What is the most adequate treatment strategy to manage malignant ascites in patients with platinum refractory advanced ovarian cancer after multiple lines of systemic therapy in areas of severe resources limitations?	Supportive care with no intervention	Sequential paracentesis for symptoms relieve	Paracentesis for drain leaving	Diuretics	Diuretic and paracentesis	Palliative intraperitoneal chemotherapy infusion	Peritoneovenous shunt	Abstain	Unqualified to answer
	0%	48%	36%	0%	10%	6%	0%	0%`	0%
What is the most adequate treatment strategy to manage obstructive uropathy in patients with platinum refractory advanced ovarian cancer after multiple lines of systemic therapy in areas of severe resources limitations?	Percutaneous nephrostomy or cystostomy depending on the level of the obstruction, and	Ureteric stenting and bladder catheter	Supportive care	Abstain	Unqualified to answer	-	-	-	-

	bladder catheter								
	38%	37%	25%	0%	0%	-	-	-	-

Supplementary Table 9. Staging and follow up

Question	Answers and frequency of responses								
What is the minimum acceptable tools for staging in patients with clinical stage **I-II** disease in an area with severe limited resources?	Operative staging only	CA 125	1 + 2	1 + 2 + Pelvic and abdominal ultrasound and chest X-ray	Abstain	Unqualified to answer	-	-	-
	23%	0%	3%	71%	3%	0%	-	-	-

What is the minimum acceptable tools for staging in patients with clinical stage III-IV disease in an area with severe limited resources?	Operative staging only	CA 125	1 + 2	1 + 2 + Pelvic and abdominal ultrasound and chest X-ray	Abstain	Unqualified to answer	-	-	-
	13%	0%	0%	87%	0%	0%	-	-	-
What's the minimum acceptable frequency of follow-up for stage I-II ovarian cancer patients after curative treatment in an area with severe resources limitations?	Every 3 months in the first 2 years, after that, every six months until 5 years from the treatment	Every 6 months until 5 years from the treatment	Annually until 5 years from the treatment	Every 6 months in the first 2 years, after that, annually until 5 years from the treatment	None until the presence of symptoms	Abstain	Unqualified to answer	-	-

	61%	21%	0%	15%	3%	0%	0%	-	-
What is the minimum acceptable tools for follow up in patients with stage I-II disease after curative treatment in an area with severe limited resources?	Clinical examination only	CA 125	1 + 2	Pelvic and abdominal ultrasound and chest X-ray + clinical examination	Pelvic and abdominal ultrasound and chest X-ray + CA 125 + + clinical examination	Pelvic and abdominal CT and chest X-ray + clinical examination	Pelvic and abdominal CT and chest X-ray + CA 125 + clinical examination	Abstain	Unqualified to answer
	9%	3%	49%	6%	18%	3%	12%	0%`	0%
What is the minimum acceptable tools for follow up in patients with stage I-II disease after curative treatment in an area with severe limited resources if computed tomography is not available?	Clinical examination only	CA 125	1 + 2	Pelvic and abdominal ultrasound and chest X-ray + clinical examination	Pelvic and abdominal ultrasound and chest X-ray + CA 125 + + clinical examination	Clinical examination only	Abstain	Unqualified to answer	-
	7%	4%	48%	14%	10%	17%	0%	0%	-

What's the minimum acceptable frequency of follow-up for stage III-IV ovarian cancer patients after curative treatment in an area with severe resources limitations?	Every 3 months in the first 2 years, after that, every six months until 5 years from the treatment	Every 6 months until 5 years from the treatment	Annually until 5 years from the treatment	Every 6 months in the first 2 years, after that, annually until 5 years from the treatment	None until the presence of symptoms	Abstain	Unqualified to answer	-	-
	85%	7%	0%	4%	4%	0%	0%	-	-
What is the minimum acceptable tools for follow up in patients with stage III-IV disease after curative treatment in an area with severe limited resources?	Clinical examination only	CA 125	1 + 2	Pelvic and abdominal ultrasound and chest X-ray + clinical examination	Pelvic and abdominal ultrasound and chest X-ray + CA 125 + + clinical examination	Pelvic and abdominal CT and chest X-ray + clinical examination	Pelvic and abdominal CT and chest X-ray + CA 125 + clinical examination	Abstain	Unqualified to answer

	3%	0%	35%	0%	45%	7%	10%	0%	0%
What is the minimum acceptable tools for follow up in patients with stage III-IV disease after curative treatment in an area with severe limited resources if computed tomography is not available?	Clinical examination only	CA 125	1 + 2	Pelvic and abdominal ultrasound and chest X-ray + clinical examination	Pelvic and abdominal ultrasound and chest X-ray + CA 125 + + clinical examination	Clinical examination only	Abstain	Unqualified to answer	-
	4%	0%	41%	4%	22%	29%	0%	0%	-
What's the minimum acceptable frequency of follow-up for stage III-IV recurrent ovarian cancer patients after response to therapy in an area with severe resources limitations?	Every 3 months in the first 2 years, after that, every six months until 5 years from	Every 6 months until 5 years from the treatment	Annually until 5 years from the treatment	Every 6 months in the first 2 years, after that, annually until 5 years from the treatment	None until the presence of symptoms	Abstain	Unqualified to answer	-	-

	the treatment								
	77%	15%	0%	4%	4%	0%	0%	-	-
What is the minimum acceptable tools for follow up in patients with recurrent ovarian cancer patients after response to therapy in an area with severe limited resources?	Clinical examination only	CA 125	1 + 2	Pelvic and abdominal ultrasound and chest X-ray + clinical examination	Pelvic and abdominal ultrasound and chest X-ray + CA 125 + + clinical examination	Pelvic and abdominal CT and chest X-ray + clinical examination	Pelvic and abdominal CT and chest X-ray + CA 125 + clinical examination	Abstain	Unqualified to answer
	4%	0%	56%	0%	18%	7%	15%	0%	0%
What is the minimum acceptable tools for follow up in patients with recurrent ovarian cancer patients after response to therapy in an area with severe limited resources if	Clinical examination only	CA 125	1 + 2	Pelvic and abdominal ultrasound and chest X-ray + clinical examination	Pelvic and abdominal ultrasound and chest X-ray + CA 125 + + clinical examination	Clinical examination only	Abstain	Unqualified to answer	-

computed tomography is not available?	4%	0%	42%	12%	21%	21%	0%	0%	-
